# Industry influence on mental health research: depression as a case example

**DOI:** 10.3389/fmed.2023.1320304

**Published:** 2024-01-23

**Authors:** Lisa Cosgrove, Elissa H. Patterson, Harold J. Bursztajn

**Affiliations:** ^1^Department of Counseling & School Psychology, University of Massachusetts, Boston, MA, United States; ^2^Departments of Psychiatry and Neurology, Institute for Healthcare Policy & Innovation, University of Michigan Medical School, Ann Arbor, MI, United States; ^3^Department of Psychiatry, Harvard Medical School, Boston, MA, United States

**Keywords:** commercialization of healthcare, industry influence in psychiatry, treatment resistant depression, antidepressant medications, social determinants of health, wellbeing, conflicts of interest, clinical practice guidelines

## Abstract

Emotional distress has been rising since before the COVID-19 pandemic and the public is told that depression is a major public health problem. For example, in 2017 depressive disorders were ranked as the third leading cause of “years lost to disability” and the World Health Organization now ranks depression as the single largest contributor to global disability. Although critical appraisals of the epidemiological data raise questions about the accuracy of population-based depression estimates, the dominance of the medical model and the marketing of psychotropics as “magic bullets,” have contributed to a dramatic rise in the prescription of psychiatric drugs. Unfortunately, the pharmaceutical industry’s influence on psychiatric research and practice has resulted in over-estimates of the effectiveness of psychotropic medications and an under-reporting of harms. This is because the principles that govern commercial entities are incongruent with the principles that guide public health research and interventions. In order to conduct mental health research and develop interventions that are in the public’s best interest, we need non-reductionist epistemological and empirical approaches that incorporate a biopsychosocial perspective. Taking depression as a case example, we argue that the socio-political factors associated with emotional distress must be identified and addressed. We describe the harms of industry influence on mental health research and show how the emphasis on “scaling up” the diagnosis and treatment of depression is an insufficient response from a public health perspective. Solutions for reform are offered.

## 1 Introduction

For more than two decades, researchers, clinicians, and policy makers have raised concerns about the commercialization of medicine (1, 2). Critics have charged that the medical profession’s culture and its public health mission are being undermined by the pharmaceutical industry’s wide-ranging influence (3). The field of psychiatry is no exception and has been the subject of numerous public and professional initiatives questioning practice as usual (4). As one prominent psychiatrist noted, the field is suffering from a “crisis of credibility” (5).

For instance, there are numerous effective non-pharmacological interventions for depression and meta-analyses of antidepressant trials have shown that on average there is a questionable risk/benefit ratio for antidepressant medication for most levels of depression (6–9). Despite this, some pharmaceutical companies have built a multi-billion-dollar global depression “market” for antidepressant medications (ADM) (10–11). Pharmaceutical companies are incentivized to uphold a biomedical understanding of distress for which they can develop and sell psychotropics and medical devices. It is thus not surprising that a 2020 study found that 7 of 10 top pharmaceutical companies spent more on sales and marketing than on research and development (12). Psychiatry as a field is also strengthened by the maintenance of the dominant narrative that promotes biomedical interventions. The dissemination of the biomedical model of depression has been successful, and the cost of this success is that it deflects attention away from the social determinants of health (SDoH). In this article we describe how industry influence and guild interests undermine psychiatry’s public health mission. We offer suggestions for developing non-reductionist epistemological and empirical approaches that synthesize the psychological and social with the biological dimensions of health and illness.

## 2 Societal distress qua disease

It has been well documented that there are increasing levels of societal despair, stress, burnout, and job dissatisfaction (13–18). However, psychiatric models codify despair, dissatisfaction with life, and accompanying physical symptoms as major depressive disorder, typically described as a biologically-based disorder that requires medication. Despite increasing treatment expenditures based on this biomedical model and the expectation of neuroscientific breakthroughs, rates of depression and well-being have not improved (19). It is clear that the serotonin hypothesis and the more general chemical imbalance theory of depression, while longstanding and historically significant, have become outdated (20). Although these theories are still embedded to some degree in the mental health field, they have not been the central focus of scientific research for more than a decade. There is growing recognition that depression is not a homogeneous condition and is thus influenced by systemic, psychosocial and biological factors (e.g., from inflammation to mitochondrial dysfunction). Nonetheless, an overly reductionist, pseudo biologically-focused approach to depression research, which focuses mainly on pharmacological interventions, obscures the connection between social injustice and emotional distress. It also fuels a belief in ‘magic bullets,’ and undermines an appreciation for the etiological complexity of mental health conditions.

Indeed, the biomedical disease model dominates clinical practice and research agendas (21) and billions of dollars of public money have been spent on these agendas. Yet, in psychiatry a focus on biology is all too often equated with genetic reductionism, which not only denies epigenetic complexity but also reinforces the status quo research agenda (22). Moreover, such reductionism has contributed to demoralization and burnout of psychiatrists and other clinicians during and after COVID and as each new wave threatens (23). Tom Insel, MD, the former director of the US National Institute of Mental Health, has been vocal about the pitfalls of reductionist biomedical research in mental health. In a statement reflecting on his leadership of the institute, he wrote, “…I think I succeeded at getting lots of really cool papers published by cool scientists at fairly large costs—I think $20 billion—I do not think we moved the needle in reducing suicide, reducing hospitalizations, improving recovery for the tens of millions of people who have mental illness” (24, 25).

## 3 Foundations of wellbeing

To better understand societal distress and depression, we need a less reductionist epistemological framework, one that considers the SDoH distress. The SDoH refer to the environmental, contextual and socio-political causes of ill health such as poverty, food or housing insecurity, inequality, and structural racism (see, e.g., https://www.who.int/health-topics/social-determinants-of-health#tab=tab_1). For example, how do poverty, institutionalized racism, and socio-economic policies that reinforce inequality, challenge wellbeing? These systemic social forces act on an individual’s ability to function. The field of lifestyle medicine helps us understand the basic needs of an individual in terms of pillars of health: restorative sleep, physical movement, plant-based nutrition, social connection, stress-management, meaning and purpose, and avoiding harmful drug use (26–28). All of these pillars are influenced by socio-environmental context, to which the biomedical disease model pays scant attention.

The effects of SDoH are profound: social and economic policies have been associated with higher suicide rates in multiple countries (29). For example, in Punjab, India, researchers found an association between alarmingly high suicide rates in farmers and higher debt burdens. They recommended that in order to decrease these suicide rates, policymakers must go beyond advocating for canonical (and intra-individual) mental health treatments. Specifically, the researchers recommended policy changes that would “stabilize the price of cash crops and relieve indebted farmers” (30). A recent review of the political and economic factors that are predictive of suicide found similar results; researchers noted that two of the strongest predictors are unemployment and low socio-economic status. In fact, research has consistently shown that increasing the minimum wage lowers suicide rates (31). Kaufman and colleagues estimated that in the US, raising the minimum wage by just USD $1.00 above the levels from 1990 to 2015 would have saved 27,550 suicide deaths (32) (see also, 33). Such findings are why the former United Nations Special Rapporteur, psychiatrist Dainius Puras, called for addressing the social determinants of health rather than simply “scaling up” the diagnosis and treatment of depression based on prevailing reductionist approaches (34, 35).

## 4 Conflicts of interest in depression research and the consequences of commercialized science

The growth of pharmacological treatment for depression, coupled with the increase in rates of depression, illustrates the confluence of commercial and guild interests in conflict with public health needs (36). During the last three decades, the American Psychiatric Association (APA), the publisher of the *Diagnostic and Statistical Manual of Mental Disorders* (*DSM*), has broadened definitions of mental illness by including new and controversial disorders and by modifying the symptom criteria for some of the mood disorders (37). Since the publication of DSM III in 1980, the *DSM* has been criticized for broadening definitions so widely that otherwise normative (albeit painful) human experiences of distress, such as bereavement, are now diagnosable. A combination of regulatory and market forces further drives diagnostic expansion. In the US and New Zealand, for example, where direct to consumer advertising (DTCA) is allowed, companies can advertise prescription pharmaceuticals only to treat specifically approved diseases. Some pharmaceutical companies have heavily marketed antidepressants via DTCA, and they have paid psychiatrists to present marketing material to primary care physicians to promote the sales of antidepressants (38). These ads and marketing campaigns have been successful: a recent study found that 80% of people believe that depression is caused by a chemical imbalance which ADM can correct (20).

Clearly, the medicalization of depression promoted by some in the pharmaceutical industry and reinforced by organized psychiatry creates a demand for developing new psychotropics. Innovation in this area in and of itself is not a bad thing. However, one of the epistemic consequences of academic-industry relationships is that they foster reductionist approaches and deflect attention away from addressing the upstream causes of ill-health. The fact that the majority of DSM IV, DSM 5 and DSM 5 TR panel members had financial ties to the manufacturers of psychotropic medications used to treat the disorders described in the manual is problematic from a public health perspective: industry is able to capitalize on the widening of diagnostic boundaries (39). For example, “Prolonged grief disorder” is a new DSM 5 TR diagnosis and there is a clinical trial assessing the efficacy of naltrexone to treat “PGD” (40). The rationale for this trial is that the researchers are conceptualizing PGD as an addiction, a disorder of the “reward system” in the brain. This conceptualization is clearly problematic from an ethical and person-centered perspective. Additionally, if naltrexone (currently off-patent) was given regulatory approval for PGD, this also would allow Mallinckrodt (the manufacturer) to significantly raise the price of this drug.

## 5 Commercial “research” for the purpose of selling products differs from scientific research

Scientific research for the purpose of the advancement of knowledge and public good adheres to rigorous ethical and experimental principles. For-profit pharmaceutical companies follow a different set of principles. In fact, publicly traded pharmaceutical companies are legally responsible for serving the best interests (including financial) of their shareholders, not for ensuring that their business promotes patient welfare or public health. It is therefore not surprising that when pharmaceutical companies sponsor research, there can be a bias toward finding and publishing data that shows the medication is safe and effective. This common bias in favor of industry products has been referred to as the “funding effect,” and it appears in different forms. For example, researchers have found that there was good concordance between results and conclusions when authors of meta-analyses had financial ties to non-profit groups. However, concordance was poor (and biased in favor of industry) in meta-analyses when the researchers had financial ties to pharmaceutical firms (41). Sismundo (42) refers to the corporate capture of the scientific literature as “ghost-management.” Relatedly, a recent scoping review that examined internal company documents found that industry used “dynamic ghost-management strategies… to safeguard their corporate interest” (43).

There are numerous ways in which for-profit companies spin “research” to sell products (44). One of the ways companies have controlled narratives about their products is to restrict access to the results of their research. Through Freedom of Information Act (FOIA) requests for data and Food and Drug Administration data, researchers interested in protecting the public (aided by a non-profit initiative known as “Restoring Invisible and Abandoned Trials” or RIAT) have begun to gain access to and reanalyze old data sets. One such reanalysis by Le Noury et al. revealed major problems in SmithKline Beecham’s “Study 329” about the treatment of adolescents with the ADM paroxetine (45). Initial publications had concluded that this ADM was safe and effective for adolescents. However, the reanalysis by LeNoury and colleagues revealed a serious public health problem—a previously unreported association between paroxetine and adverse events, including suicidal ideation and behavior.

## 6 Industry and guild conflicts of interest get codified in clinical practice guidelines

Clinical Practice guidelines (CPGs) are understood to be an essential part of evidence-based medicine. Unfortunately, many CPGs are untrustworthy, in part, because many guideline development groups are implicitly influenced by guilds and industry sources (46). The problem is so pernicious that some researchers have called for a moratorium on guidelines produced by specialty groups, and the Institute of Medicine (now Academy of Medicine) maintains that financial conflict of interest disclosure is not enough—guideline developers should be free of industry ties (47). Furthermore, the number of research papers and guidelines circulating in the medical literature makes it virtually impossible for busy clinicians to identify which ones are trustworthy and relevant. Researchers who assessed the quality of APA’s influential guideline on the treatment of depression found that fewer than half (44.4%) of the studies supporting the recommendations met criteria for high quality (46). They also found that all of the authors of the guideline had ties to pharmaceutical companies that manufacture antidepressants. Perhaps not surprisingly, this guideline recommended antidepressants (ADM) for all levels of depression, including mild depression. Such a recommendation runs counter to the evidence; there is ongoing debate about the details, but on average, drug-placebo differences are reported to be small and not clinically meaningful for most individuals except those with the most severe forms of depression (6–9, 48).

There is increasing evidence that ADM are not the “magic bullets” that some might have hoped for. And still, despite clinical trial evidence and a growing awareness of the limits of a narrow focus on neurotransmitters, the dominant paradigm in biomedical depression research and treatment is to label a person as having “treatment resistant depression (“TRD”), if a person does not respond to ADM (49). The use of this term and acronym is problematic for many reasons, not the least of which is that there is no consensually agreed upon definition of TRD (e.g., how many ADMs must be tried or whether psychotherapy or other interventions should be tried before applying the label) (50). Through its reductionist focus on ADM, TRD perpetuates the misconception, codified in some CPGs for depression, that there is a good risk/benefit ratio for ADM for all levels of depression. Despite the fact that a PubMed search for “treatment-resistant depression” yields over 7,900 articles, TRD is increasingly recognized as a methodologically flawed and heterogenous research category (50–54). Unfortunately, despite significant questions about the validity of TRD, this construct is still used to justify research and patient care with treatments whose harms may outweigh the benefits over the long-term (e.g., ketamine infusions) (51). For example, even with concerns about side effects, adverse events and the long-term effectiveness of ketamine, a recent business report described the exponential rise of ketamine clinics (55). It reported that in the U.S., the market size of ketamine clinics was valued at over USD 3 billion in 2022 and stated that further growth “is expected to be driven primarily by the increasing prevalence of major depressive disorder” (56). Ketamine research exemplifies the pharmaceutical industry’s influence on reductionist research agendas that in some cases promote financial gain over public health interests.

However, it is important to note that the increasing interest in alternative treatments like ketamine stems not only from the conceptualization of “TRD”, but also from the pursuit of a wider array of interventions for patients who do not respond to current therapies, including non-pharmacological ones. These patients highlight the need for both novel treatments as well as the need for greater attention to the upstream causes of distress.

## Summary recommendations

1. Medical journal editors should be free of industry ties. Relatedly, *publicly sponsored* Health Technology Assessment entities, such as the National Institute for Health and Care Excellence (N.I.C.E.), should play a central role in evaluating healthcare interventions.
2. To enhance shared-decision-making, it is important to conceptualize informed consent as a process, not a one-time or proforma event.
3. Knowledge about the SDoH, the importance of epistemic humility, and critical thinking are essential aspects of clinical training.
4. Robust international public health campaigns—ones that disseminate accurate and balanced information about the problems of widening diagnostic boundaries and industry-funded research—are sorely needed.
5. Regulatory bodies should require head-to-head comparisons of randomized controlled trials for comparisons for ADM.
6. In order to broaden rights-based approaches to mental health, it is critical that a diverse group of professionals and people with lived experience be included in mental health research and policy making.

## 7 Recommendations and discussion

The role of biological factors in the etiology of depression needs to continue to be investigated. Intra-individual treatments, including but not limited to psychotropics, also need to be a part of population based mental health interventions. However, medicine is most effectively practiced when it is guided by a biopsychosocial model of preventing and treating illness-related suffering and impairment (57). Psychiatry can best be understood as a biopsychosocial practice of alleviating certain forms of suffering. The understanding that informs medical and psychiatric practice is in part biological, but biological processes occur in a psychological and social context. Similarly, medications can be part of treatment, but not the whole of it. Medication effects themselves are a product not only of a biochemical substance, but also of the patient’s mental set within a physical and social setting (58, 59). Also, the fact that some patients do not respond to pharmacotherapy or psychotherapy has spurred the development of randomized clinical trials for using dietary approaches to treating depression and anxiety (e.g., the Mediterranean and ketogenic diets). Lack of a significant response to traditional interventions is also a driver in the emergence of “the third wave” of cognitive behavioral therapies such as Dialectical Behavior Therapy (DBT) and Mindfulness-Based Cognitive Therapy (MBCT).

Indeed, it is widely acknowledged that social, lifestyle. and environmental factors significantly influence well-being (31, 60–63). Yet, because of the dominance of the biomedical model in the mental health field, the social determinants of mental health and their interactions with the basic pillars of health get short shrift. As we have shown here, this is due in part to commercial and guild interests that converge to create a climate in which billions of dollars are spent on researching and treating human suffering as a disorder. Of course, even if it were possible to eliminate all industry influence on diagnostic and clinical practice guidelines, the upstream causes of ill-health would still be left unaddressed. We offer the following recommendations as non-reductionist epistemological and empirical approaches that can help enhance the quality of depression research and clinical care guidelines.

To provide clinicians with information that facilitates high quality care, we need to establish trustworthy processes for evaluating health technology independent of industry conflicts of interest (64). The peer review process is not robust enough to prevent publication bias and disclosure of FCOI cannot protect against implicit bias. Therefore, we recommend that the International Committee of Medical Journal Editors^1^ mandate that medical journal editors be free of industry ties. In addition, we recommend maintaining publicly sponsored Health Technology Assessment entities, such as the National Institute for Health and Care Excellence (N.I.C.E.), that evaluate healthcare interventions to inform clinical practice and policymaking.

In medicine there is a growing awareness of the limits of a paternalistic approach, an awareness that promotes compassionate dialogue and a more person-centered approach to patient care. When psychotropic medication is indicated, it should be prescribed in a manner that respects the patient’s dignity. The prescriber needs to talk with the patient in a meaningful manner. In light of the fact that 13% of US adults take a prescribed ADM (65, 66), informed consent is a critical issue. Thus, it is important to conceptualize informed consent as a process, not a one-time or proforma event (67). A crucial aspect of this process is presenting and discussing meaningful treatment alternatives based on the patient’s values and context.

One important way in which the field of psychiatry can adopt a posture of epistemic and cultural humility is to explicitly acknowledge the limits of our knowledge about western biomedical interventions. For example, the fact that we do not know exactly how ADM or other biomedical psychiatric treatments for depression “work” –nor can we predict for whom—should be a standard part of shared decision-making when ADM is being considered as a treatment option.

We need to encourage health care professionals and the public to think critically about industry friendly conceptualizations such as “treatment resistant depression/TRD” and “prolonged grief disorder.” For example, TRD is a heterogenous category that lacks diagnostic validity. It should not be used to justify treatment or research with risk/benefit ratios that would otherwise be considered unacceptable. Non-profit organizations such as the Lown Institute (ref https://lowninstitute.org/) and https://rxbalance.org/ are excellent examples of independent organizations that encourage critical thinking and provide balanced and accurate information about health-related issues.

The Global Mental Health Movement (68, 69) has been dominated by a Western biomedical approach (34, 70) that has not been as effective as originally hoped in the places where it is already prominent (71). We need a robust international public health campaign that disseminates accurate and balanced information about the problems of widening diagnostic boundaries and industry-funded research. And even more importantly, accurate, non-biased information about the many effective low-risk strategies to promote wellbeing need to be offered as an antidote to medicalization. The British Medical Journal’s “Too Much Medicine” initiative and the “Restoring Invisible and Abandoned Trials” initiative are helpful examples of how to promote the dissemination of accurate and balanced information about overdiagnosis, overtreatment, and the efficacy of psychotropics.

Regulatory bodies should require head-to-head comparisons of randomized controlled trials for ADM to avoid the approval of more expensive, marginally effective “me-too” drugs (If ADM is as efficacious as industry claims, it is a violation of the principle of equipoise to conduct only placebo-controlled trials).

Although healthcare professionals cannot be expected to single-handedly address social and environmental factors during medical visits. There is a pressing need to develop healthcare curricula that integrate the social determinants of health. For example, medical-legal partnerships (MLPs) are increasingly being developed in healthcare settings. MLPs have a *pro-bono* attorney on site who can address the “health-harming legal needs” (e.g., immigration status; unsafe housing) of patients with mental health issues. Additionally, the structural competency movement, https://structuralcompetency.org/ is another resource that educates clinicians in training about individual and policy level interventions that address the effects of structural racism and how inequality negatively impacts mental health.

In order to broaden rights-based approaches to depression treatment and mental health more generally, it is critical that a diverse group of professionals and people with lived experience be included in mental health research and policy making.

## Data availability statement

The original contributions presented in the study are included in the article/supplementary material, further inquiries can be directed to the corresponding author.

## Author contributions

EP: Conceptualization, Writing – original draft, Writing – review & editing. LC: Conceptualization, Writing – original draft, Writing – review & editing. HB: Conceptualization, Writing – review & editing.

## Footnotes

^1^https://www.icmje.org/
